# 2-Carboxy­anilinium chloride monohydrate

**DOI:** 10.1107/S1600536808029267

**Published:** 2008-09-17

**Authors:** Syed Azeem Rashid Ali Zaidi, M. Nawaz Tahir, Javed Iqbal, Muhammad Ashraf Chaudhary

**Affiliations:** a Institute of Chemistry, University of the Punjab, Lahore 54590, Pakistan; b Department of Physics, University of Sargodha, Sagrodha, Pakistan; c Department of Chemistry, F.C. College & University, Lahore, Pakistan

## Abstract

In the mol­ecule of the title compound, C_7_H_8_NO_2_^+^·Cl^−^·H_2_O, an intra­molecular N—H⋯O hydrogen bond results in the formation of a non-planar six-membered ring adopting a flattened boat conformation. In the crystal structure, inter­molecular O—H⋯O and N—H⋯Cl hydrogen bonds link the mol­ecules. There is a C=O⋯π contact between the carbonyl unit and the centroid of the benzene ring. There is a C=O⋯π contact [C⋯*Cg* = 3.5802 (18), C—O⋯*Cg* = 89 (1)°] between the carbonyl unit and the centroid of the benzene ring.

## Related literature

For applications of anthranilic acid derivatives, see: Congiu *et al.* (2005 ▶); Nittoli *et al.* (2005 ▶). For a related structure, see: Bahadur *et al.* (2007 ▶); For bond-length data, see: Allen *et al.* (1987 ▶). For ring puckering parameters, see: Cremer & Pople (1975 ▶).
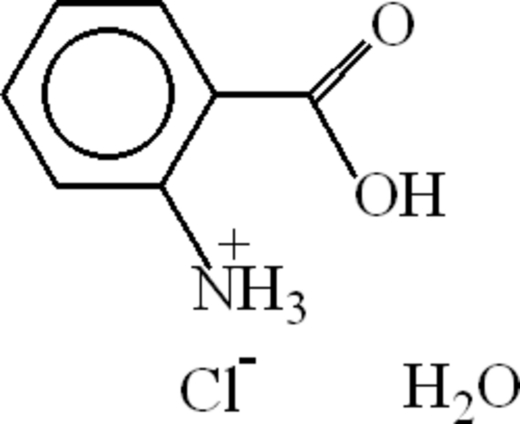


## Experimental

### 

#### Crystal data

C_7_H_8_NO_2_^+^·Cl^−^·H_2_O
*M* *_r_* = 191.61
Monoclinic, 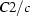

*a* = 23.094 (4) Å
*b* = 4.7833 (8) Å
*c* = 16.381 (3) Å
β = 91.605 (9)°
*V* = 1808.8 (5) Å^3^
*Z* = 8
Mo *K*α radiation
μ = 0.39 mm^−1^
*T* = 296 (2) K
0.28 × 0.10 × 0.06 mm

#### Data collection

Bruker Kappa APEXII CCD diffractometer
Absorption correction: multi-scan (*SADABS*; Bruker, 2005 ▶) *T*_min_ = 0.975, *T*_max_ = 0.985
9747 measured reflections
2238 independent reflections
1749 reflections with *I* > 2σ(*I*)
*R*_int_ = 0.025

#### Refinement

*R*[*F* ^2^ > 2σ(*F* ^2^)] = 0.036
*wR*(*F* ^2^) = 0.101
*S* = 1.05
2238 reflections
139 parameters
H atoms treated by a mixture of independent and constrained refinement
Δρ_max_ = 0.30 e Å^−3^
Δρ_min_ = −0.22 e Å^−3^

### 

Data collection: *APEX2* (Bruker, 2007 ▶); cell refinement: *APEX2*; data reduction: *SAINT* (Bruker, 2007 ▶); program(s) used to solve structure: *SHELXS97* (Sheldrick, 2008 ▶); program(s) used to refine structure: *SHELXL97* (Sheldrick, 2008 ▶); molecular graphics: *ORTEP-3 for Windows* (Farrugia, 1997 ▶) and *PLATON* (Spek, 2003 ▶); software used to prepare material for publication: *WinGX* publication routines (Farrugia, 1999 ▶) and *PLATON*.

## Supplementary Material

Crystal structure: contains datablocks text, I. DOI: 10.1107/S1600536808029267/hk2527sup1.cif

Structure factors: contains datablocks I. DOI: 10.1107/S1600536808029267/hk2527Isup2.hkl

Additional supplementary materials:  crystallographic information; 3D view; checkCIF report

## Figures and Tables

**Table 1:** Hydrogen-bond geometry (Å, °)

*D*—H⋯*A*	*D*—H	H⋯*A*	*D*⋯*A*	*D*—H⋯*A*
O1—H1⋯O3^i^	0.82 (2)	1.73 (2)	2.539 (2)	171 (2)
N1—H1*A*⋯O2	0.91 (2)	1.91 (2)	2.6820 (19)	142.1 (18)
N1—H1*B*⋯Cl1^ii^	0.88 (2)	2.28 (2)	3.1464 (16)	166.6 (18)
N1—H1*C*⋯Cl1^iii^	0.958 (19)	2.181 (19)	3.1231 (16)	167.5 (18)
O3—H3*A*⋯Cl1	0.76 (3)	2.42 (3)	3.1452 (18)	160 (3)
O3—H3*B*⋯O2^iv^	0.78 (3)	2.04 (3)	2.777 (3)	159 (3)

Symmetry codes: (i) 
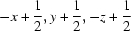
; (ii) 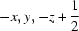; (iii) 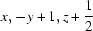; (iv) 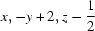.

**Fig. 1.**
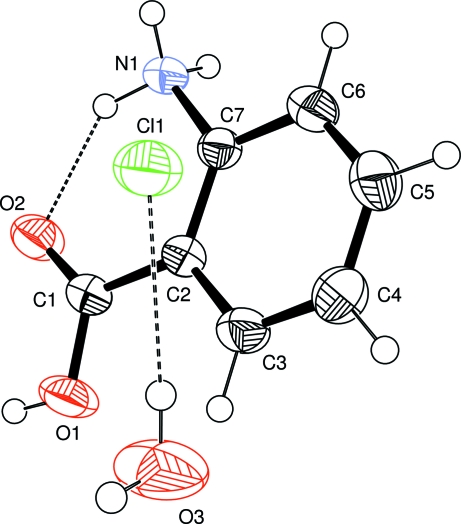
The molecular structure of the title molecule, with the atom-numbering scheme. Displacement ellipsoids are drawn at the 50% probability level. Hydrogen bonds are shown as dashed lines.

**Fig. 2.**
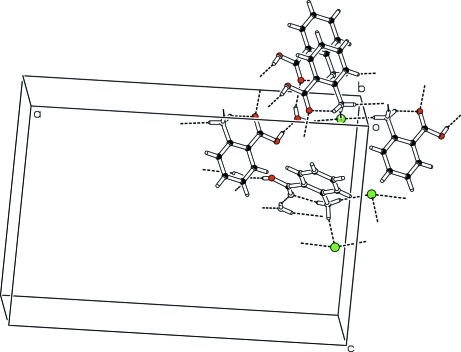
A packing diagram of the title compound. Hydrogen bonds are shown as dashed lines.

## supplementary crystallographic information

### Comment

Anthranilic acid is widely used for various purposes such as production of dyes, pigments and saccharin. Its derivatives also have importance in medicinal chemistry and are used as antiinflammatory and anticancer agents (Congiu *et al.*,  2005) and for inhibition of Hepatitis C NS5B polymerase (Nittoli *et al.*,  2005). The title compound has been prepared to see its bioactivity with hope that it will be a good antibactarial agent at the economical cost and to utilize for preparing further derivatives. Crystal structures of proton transfer compound of 2-aminobenzoic acid with nitric acid (Bahadur *et al.*,  2007) has been reported, where the intramolecular N-H···O hydrogen bond has also been observed.

In the molecule of the title compound (Fig. 1), the bond lengths (Allen *et al.*,  1987) and angles are within normal ranges. Ring A (C2-C7) is, of course, planar. The intramolecular N-H···O hydrogen bond (Table 1) results in the formation of a nonplanar six-membered ring B (N1/O2/C1/C2/C7/H1A) having total puckering amplitude, Q_T_, of 0.127 (3) Å, and flattened boat conformation [φ = -21.25 (3)°, θ = 44.41 (3)°] (Cremer & Pople, 1975).

In the crystal structure, intermolecular O-H···O and N-H···Cl hydrogen bonds (Table 1) link the molecules (Fig. 2), in which they may be effective in the stabilization of the structure. The C=O···π contact (Table 1) between the carbonyl moiety and the centroid of the benzene ring may further stabilize the structure.

### Experimental

For the preparation of the title compound, 2-aminobenzoic acid (4.11 g, 3 mmol) and trichloroacetic acid (1.64 g, 1 mmol) were neutrilized with Na_2_CO_3_ separately in H_2_O, and then mixed together. Na_2_CO_3_ (3.18 g) was added to the resulting mixture and refluxed for 4 h. The refluxed solution was cooled, and concenterated HCl was added to get PH = 1. The solution was kept in open air for 3 d to get the suitable crystals.

### Refinement

H atoms were located in difference syntheses and refined as [O-H = 0.82 (2) Å (for OH); O-H = 0.76 (3) and 0.78 (3) Å (for H_2_O); N-H = 0.88 (2)-0.958 (19) Å, ( for NH_3_) and C-H = 0.93 (2)-0.96 (2) Å (for CH)] and constrained to ride on their parent atoms with U_iso_(H) = xU_eq_(C,N,O), where x = 1.5 for NH_3_ H and x = 1.2 for all other H atoms.

### Crystal data

**Table:**

C_7_H_8_NO_2_^+^·Cl^−^·H_2_O	*F*(000) = 800
*M**_r_* = 191.61	*D*_x_ = 1.407 Mg m^−^^3^
Monoclinic, *C*2/*c*	Mo *K*α radiation, λ = 0.71073 Å
Hall symbol: -C 2yc	Cell parameters from 2238 reflections
*a* = 23.094 (4) Å	θ = 2.5–28.3°
*b* = 4.7833 (8) Å	µ = 0.39 mm^−^^1^
*c* = 16.381 (3) Å	*T* = 296 K
β = 91.605 (9)°	Prism, brown
*V* = 1808.8 (5) Å^3^	0.28 × 0.10 × 0.06 mm
*Z* = 8	

### Data collection

**Table:**

Bruker Kappa APEXII CCD diffractometer	2238 independent reflections
Radiation source: fine-focus sealed tube	1749 reflections with *I* > 2σ(*I*)
graphite	*R*_int_ = 0.025
Detector resolution: 7.5 pixels mm^-1^	θ_max_ = 28.3°, θ_min_ = 2.5°
ω scans	*h* = −30→30
Absorption correction: multi-scan (SADABS; Bruker, 2005)	*k* = −6→3
*T*_min_ = 0.975, *T*_max_ = 0.985	*l* = −21→20
9747 measured reflections	

### Refinement

**Table:**

Refinement on *F*^2^	Primary atom site location: structure-invariant direct methods
Least-squares matrix: full	Secondary atom site location: difference Fourier map
*R*[*F*^2^ > 2σ(*F*^2^)] = 0.036	Hydrogen site location: inferred from neighbouring sites
*wR*(*F*^2^) = 0.101	H atoms treated by a mixture of independent and constrained refinement
*S* = 1.05	*w* = 1/[σ^2^(*F*_o_^2^) + (0.0476*P*)^2^ + 1.0872*P*] where *P* = (*F*_o_^2^ + 2*F*_c_^2^)/3
2238 reflections	(Δ/σ)_max_ < 0.001
139 parameters	Δρ_max_ = 0.30 e Å^−^^3^
0 restraints	Δρ_min_ = −0.22 e Å^−^^3^

### Special details

**Table:**

Geometry. Bond distances, angles *etc*. have been calculated using the rounded fractional coordinates. All su's are estimated from the variances of the (full) variance-covariance matrix. The cell e.s.d.'s are taken into account in the estimation of distances, angles and torsion angles
Refinement. Refinement of *F*^2^ against ALL reflections. The weighted *R*-factor *wR* and goodness of fit *S* are based on *F*^2^, conventional *R*-factors *R* are based on *F*, with *F* set to zero for negative *F*^2^. The threshold expression of *F*^2^ > σ(*F*^2^) is used only for calculating *R*-factors(gt) *etc*. and is not relevant to the choice of reflections for refinement. *R*-factors based on *F*^2^ are statistically about twice as large as those based on *F*, and *R*- factors based on ALL data will be even larger.

### Fractional atomic coordinates and isotropic or equivalent isotropic displacement parameters (Å^2^)

**Table:**

	*x*	*y*	*z*	*U*_iso_*/*U*_eq_	
Cl1	0.05634 (2)	0.81018 (10)	0.08328 (3)	0.0425 (2)	
O1	0.24332 (5)	0.7928 (3)	0.36801 (9)	0.0551 (5)	
O2	0.17708 (5)	0.9236 (3)	0.45555 (8)	0.0493 (4)	
O3	0.18909 (7)	0.6690 (4)	0.07558 (14)	0.0796 (7)	
N1	0.07438 (6)	0.6617 (3)	0.45470 (9)	0.0340 (4)	
C1	0.19199 (7)	0.7781 (3)	0.39900 (10)	0.0338 (5)	
C2	0.15328 (6)	0.5699 (3)	0.35784 (9)	0.0304 (4)	
C3	0.17259 (8)	0.4219 (4)	0.29012 (10)	0.0408 (5)	
C4	0.13710 (9)	0.2318 (4)	0.25017 (11)	0.0462 (6)	
C5	0.08157 (8)	0.1841 (4)	0.27706 (12)	0.0466 (6)	
C6	0.06160 (7)	0.3269 (4)	0.34388 (11)	0.0398 (5)	
C7	0.09727 (6)	0.5188 (3)	0.38354 (9)	0.0300 (4)	
H1	0.2619 (10)	0.922 (5)	0.3885 (14)	0.0661*	
H1A	0.0991 (9)	0.802 (4)	0.4684 (12)	0.0510*	
H1B	0.0399 (10)	0.732 (4)	0.4423 (13)	0.0510*	
H1C	0.0724 (9)	0.534 (4)	0.4996 (12)	0.0510*	
H3	0.2113 (9)	0.452 (4)	0.2717 (11)	0.0489*	
H3A	0.1584 (14)	0.692 (7)	0.0892 (19)	0.0955*	
H3B	0.1945 (14)	0.777 (6)	0.041 (2)	0.0955*	
H4	0.1511 (9)	0.133 (5)	0.2061 (13)	0.0554*	
H5	0.0582 (9)	0.052 (4)	0.2493 (12)	0.0558*	
H6	0.0245 (9)	0.298 (4)	0.3628 (12)	0.0477*	

### Atomic displacement parameters (Å^2^)

**Table:**

	*U*^11^	*U*^22^	*U*^33^	*U*^12^	*U*^13^	*U*^23^
Cl1	0.0314 (2)	0.0499 (3)	0.0463 (3)	−0.0004 (2)	0.0035 (2)	−0.0055 (2)
O1	0.0320 (7)	0.0668 (9)	0.0673 (9)	−0.0189 (6)	0.0174 (6)	−0.0255 (7)
O2	0.0401 (7)	0.0554 (8)	0.0531 (7)	−0.0144 (6)	0.0133 (6)	−0.0206 (6)
O3	0.0408 (8)	0.0837 (13)	0.1153 (15)	0.0249 (8)	0.0207 (9)	0.0514 (11)
N1	0.0255 (7)	0.0362 (8)	0.0406 (8)	−0.0012 (6)	0.0081 (5)	−0.0030 (6)
C1	0.0283 (8)	0.0362 (8)	0.0372 (8)	−0.0030 (6)	0.0049 (6)	0.0006 (7)
C2	0.0279 (7)	0.0312 (8)	0.0323 (7)	−0.0017 (6)	0.0041 (6)	0.0009 (6)
C3	0.0357 (9)	0.0456 (10)	0.0416 (9)	−0.0040 (8)	0.0111 (7)	−0.0063 (8)
C4	0.0512 (11)	0.0487 (10)	0.0391 (9)	−0.0031 (8)	0.0077 (8)	−0.0135 (8)
C5	0.0445 (10)	0.0462 (10)	0.0486 (10)	−0.0088 (8)	−0.0048 (8)	−0.0109 (8)
C6	0.0292 (8)	0.0435 (10)	0.0466 (9)	−0.0053 (7)	0.0018 (7)	−0.0030 (8)
C7	0.0278 (7)	0.0307 (8)	0.0315 (7)	0.0005 (6)	0.0031 (6)	0.0014 (6)

### Geometric parameters (Å, °)

**Table:**

O1—C1	1.304 (2)	C2—C3	1.399 (2)
O2—C1	1.216 (2)	C2—C7	1.393 (2)
O1—H1	0.82 (2)	C3—C4	1.377 (3)
O3—H3B	0.78 (3)	C4—C5	1.387 (3)
O3—H3A	0.76 (3)	C5—C6	1.380 (3)
N1—C7	1.463 (2)	C6—C7	1.383 (2)
N1—H1B	0.88 (2)	C3—H3	0.96 (2)
N1—H1C	0.958 (19)	C4—H4	0.93 (2)
N1—H1A	0.91 (2)	C5—H5	0.94 (2)
C1—C2	1.487 (2)	C6—H6	0.93 (2)
			
Cl1···O3	3.1452 (18)	N1···O2	2.6820 (19)
Cl1···N1^i^	3.3227 (16)	C1···C3^iv^	3.581 (2)
Cl1···N1^ii^	3.1464 (16)	C1···O3^vi^	3.339 (2)
Cl1···N1^iii^	3.1231 (16)	C1···C4^iv^	3.477 (3)
Cl1···H5^iv^	2.96 (2)	C3···C1^x^	3.581 (2)
Cl1···H3A	2.42 (3)	C3···O1^viii^	3.338 (2)
Cl1···H6^v^	3.13 (2)	C4···C1^x^	3.477 (3)
Cl1···H1B^ii^	2.28 (2)	C1···H1A	2.46 (2)
Cl1···H1A^i^	2.84 (2)	H1···H3B^vi^	2.27 (4)
Cl1···H1C^iii^	2.181 (19)	H1···H3A^vi^	2.27 (4)
O1···C3^vi^	3.338 (2)	H1···O3^vi^	1.73 (2)
O1···O3^vi^	2.539 (2)	H1A···C1	2.46 (2)
O2···O3^vii^	2.777 (3)	H1A···O2	1.91 (2)
O2···N1	2.6820 (19)	H1A···Cl1^vii^	2.84 (2)
O3···O2^i^	2.777 (3)	H1B···Cl1^ii^	2.28 (2)
O3···O1^viii^	2.539 (2)	H1B···H6	2.47 (3)
O3···C1^viii^	3.339 (2)	H1C···Cl1^ix^	2.181 (19)
O3···Cl1	3.1452 (18)	H3···O1	2.372 (19)
O1···H4^vi^	2.86 (2)	H3···O1^viii^	2.655 (19)
O1···H3^vi^	2.655 (19)	H3A···O2^i^	2.90 (3)
O1···H3	2.372 (19)	H3A···H1^viii^	2.27 (4)
O2···H3A^vii^	2.90 (3)	H3A···Cl1	2.42 (3)
O2···H3B^vii^	2.04 (3)	H3B···H1^viii^	2.27 (4)
O2···H1A	1.91 (2)	H3B···O2^i^	2.04 (3)
O3···H1^viii^	1.73 (2)	H4···O1^viii^	2.86 (2)
N1···Cl1^ix^	3.1231 (16)	H5···Cl1^x^	2.96 (2)
N1···Cl1^vii^	3.3227 (16)	H6···Cl1^xi^	3.13 (2)
N1···Cl1^ii^	3.1464 (16)	H6···H1B	2.47 (3)
			
C1—O1—H1	110.7 (16)	C3—C4—C5	120.07 (17)
H3A—O3—H3B	107 (3)	C4—C5—C6	120.20 (17)
C7—N1—H1C	109.9 (12)	C5—C6—C7	119.54 (15)
H1A—N1—H1B	109.5 (18)	N1—C7—C2	121.21 (13)
H1B—N1—H1C	110.9 (19)	C2—C7—C6	121.35 (14)
C7—N1—H1A	107.7 (13)	N1—C7—C6	117.43 (13)
H1A—N1—H1C	109.0 (17)	C2—C3—H3	119.5 (11)
C7—N1—H1B	109.8 (14)	C4—C3—H3	119.7 (11)
O1—C1—O2	123.05 (15)	C5—C4—H4	120.6 (13)
O1—C1—C2	113.64 (14)	C3—C4—H4	119.3 (13)
O2—C1—C2	123.31 (15)	C4—C5—H5	118.7 (13)
C3—C2—C7	118.04 (14)	C6—C5—H5	121.1 (13)
C1—C2—C7	122.14 (13)	C5—C6—H6	121.5 (12)
C1—C2—C3	119.82 (14)	C7—C6—H6	119.0 (12)
C2—C3—C4	120.80 (17)		
			
O1—C1—C2—C3	−2.8 (2)	C3—C2—C7—N1	178.91 (14)
O1—C1—C2—C7	178.32 (14)	C3—C2—C7—C6	0.3 (2)
O2—C1—C2—C3	176.32 (16)	C2—C3—C4—C5	−0.3 (3)
O2—C1—C2—C7	−2.5 (2)	C3—C4—C5—C6	0.1 (3)
C1—C2—C3—C4	−178.82 (16)	C4—C5—C6—C7	0.3 (3)
C7—C2—C3—C4	0.1 (2)	C5—C6—C7—N1	−179.16 (16)
C1—C2—C7—N1	−2.2 (2)	C5—C6—C7—C2	−0.5 (3)
C1—C2—C7—C6	179.17 (15)		

Symmetry codes: (i) *x*, −*y*+2, *z*−1/2; (ii) −*x*, *y*, −*z*+1/2; (iii) *x*, −*y*+1, *z*−1/2; (iv) *x*, *y*+1, *z*; (v) −*x*, *y*+1, −*z*+1/2; (vi) −*x*+1/2, *y*+1/2, −*z*+1/2; (vii) *x*, −*y*+2, *z*+1/2; (viii) −*x*+1/2, *y*−1/2, −*z*+1/2; (ix) *x*, −*y*+1, *z*+1/2; (x) *x*, *y*−1, *z*; (xi) −*x*, *y*−1, −*z*+1/2.

### Hydrogen-bond geometry (Å, °)

**Table:**

*D*—H···*A*	*D*—H	H···*A*	*D*···*A*	*D*—H···*A*
O1—H1···O3^vi^	0.82 (2)	1.73 (2)	2.539 (2)	171 (2)
N1—H1A···O2	0.91 (2)	1.91 (2)	2.6820 (19)	142.1 (18)
N1—H1B···Cl1^ii^	0.88 (2)	2.28 (2)	3.1464 (16)	166.6 (18)
N1—H1C···Cl1^ix^	0.958 (19)	2.181 (19)	3.1231 (16)	167.5 (18)
O3—H3A···Cl1	0.76 (3)	2.42 (3)	3.1452 (18)	160 (3)
O3—H3B···O2^i^	0.78 (3)	2.04 (3)	2.777 (3)	159 (3)
C1—O2···Cg^iv^	1.216 (2)	3.3988 (16)	3.5802 (18)	88.5 (1)

Symmetry codes: (vi) −*x*+1/2, *y*+1/2, −*z*+1/2; (ii) −*x*, *y*, −*z*+1/2; (ix) *x*, −*y*+1, *z*+1/2; (i) *x*, −*y*+2, *z*−1/2; (iv) *x*, *y*+1, *z*.
